# Supplementary material for: Differential Effects of Furin Deficiency on Insulin Receptor Processing and Glucose Control in Liver and Pancreatic β Cells of Mice
Source: Int J Mol Sci. 2021 Jun 14;22(12):6344. doi: 10.3390/ijms22126344 (PMC8231939; doi:10.3390/ijms22126344)
Supplement: Supplementary file 1 [file ijms-22-06344-s001.zip › ijms-1255190-supplementary.pdf]

International Journal of
Molecular Sciences

MDPI

Table S1. Primer specifications for RT-qPCR.

Primer name	Forward Primer (5'-3')	Reverse Primer (5'-3')	Amplification efficiency (%)	Tm (℃)	Location (exons)	Lenght of amplicon (bp)	Final concentration (µM)
Gapdh	5'-CCCCAATGTGTCCGTCGTG-3'	5'-GCCTGCTTCACCACCTTCT-3'	91,7	60	5-6	84	2,5
Furin	5'-GAGCTGAGATCCTGGTTGCT-3'	5'-AGGTTGTGGAAGCCATGC-3'	96,3	60	2	167	2,5
Pcsk5	5'-AGGTGGAGTGGATCCAACAG-3'	5'-CCGTGTAGCCTCTCTTCCAG-3'	89	60	2-4	189	2,5
Pcsk6	5'-AGCGTGGCTACACAGGAAAG-3'	5'-TATCTCGGGGATGGGTCATA-3'	98,1	60	4-5	142	2,5
Pcsk7	5'-CCACCCTGATGAGGAGAATG-3'	5'-GCCACAGCCTCCATACTGTC-3'	86,4	54	5-6	165	2,5
Trib3	5'-TGGCTGGCAGATACCCATTC-3'	5'-CAAGTCGCTCTGAAGGTTCCTT-3'	97,9	58	4	150	2,5
Chop	5'-CCACCACACCTGAAAGCAGAA-3'	5'-AGGTGAAAGGCAGGGACTCA-3'	99,4	58	2-3	67	2,5
Atf4	5'-TCGATGCTCTGTTTCGAATG-3'	5'-AGAATGTAAAGGGGGCAACC-3'	91,8	56	3	192	2,5

Figure S1

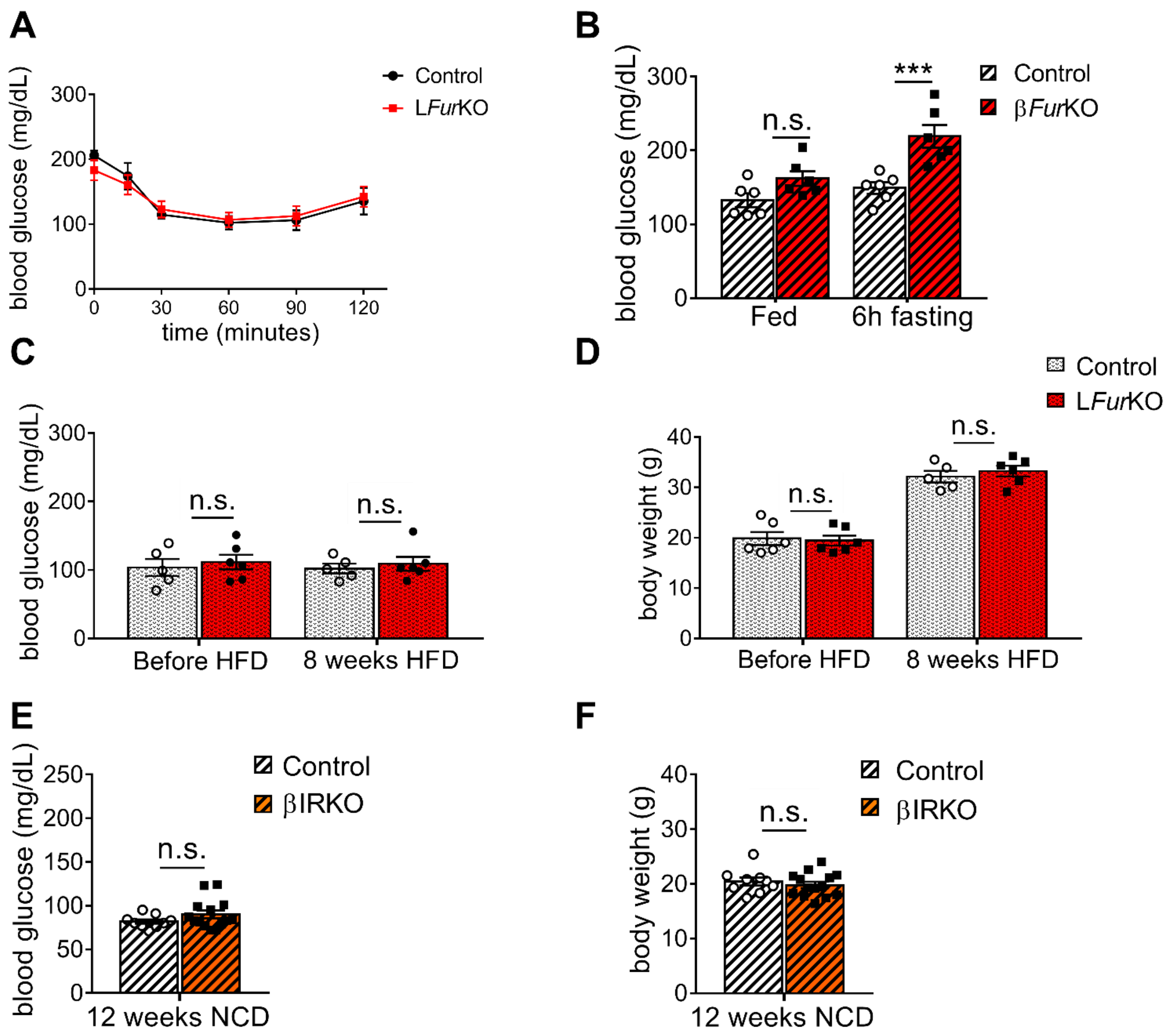

Figure S1. (A) Intraperitoneal insulin tolerance test (IPITT) on 18-week-old male L*Fur*KO and control mice fed on HFD for 10 weeks (n=5-6 mice/group). *p<0.05 determined by repeated measure two-way ANOVA. All data are presented as mean ± SEM. (B) Fed and 6h fasting blood glucose of control (*Fur*$^{fl/fl}$, RIPCre$^{-/-}$) and β*Fur*KO (*Fur*$^{fl/fl}$, RIPCre$^{+/-}$) 16-week-old male mice on HFD for 8 weeks (n=6 mice/group).***p<0.001 determined by one-way ANOVA with Sidak's multiple comparison test. (C) 16h fasting blood glucose of control (*Fur*$^{fl/fl}$, AlbCre$^{-/-}$) and L*Fur*KO (*Fur*$^{fl/fl}$, AlbCre$^{+/-}$) 8 and 16-week-old male mice before and after 8 weeks HFD (n=5-6 mice/group). No significant differences were detected by one-way ANOVA with Sidak's multiple comparison test. (D) 16h fasting body weight (g) of control (*Fur*$^{fl/fl}$, AlbCre$^{-/-}$) and L*Fur*KO (*Fur*$^{fl/fl}$, AlbCre$^{+/-}$) 8-week and 16-week-old male mice on NCD and 8 weeks HFD respectively (n=5-6 mice/group). No significant differences were detected by one-way ANOVA with Sidak's multiple comparison test. All data are represented as mean ± SEM. (E) 16h fasting blood glucose of control (IR$^{lox/lox}$, RIPCre$^{-/-}$) and βIRKO (IR$^{lox/lox}$, RIPCre$^{+/-}$) 12-week-old male mice fed on NCD (n=9-14 mice/group). No significant differences were detected by unpaired t test with Welch's correction. (F) 16h fasting body weight (g) of control (IR$^{lox/lox}$, RIPCre$^{-/-}$) and βIRKO (IR$^{lox/lox}$, RIPCre$^{+/-}$) 12-week-old male mice fed on NCD (n=9-14 mice/group). No significant differences were detected by unpaired t test. All data are represented as mean ± SEM.
